# Supplementary figures and images for: Hypopituitarism in Sox3 null mutants correlates with altered NG2-glia in the median eminence and is influenced by aspirin and gut microbiota
Source: PLoS Genet. 2024 Sep 26;20(9):e1011395. doi: 10.1371/journal.pgen.1011395 (PMC11426531; doi:10.1371/journal.pgen.1011395)

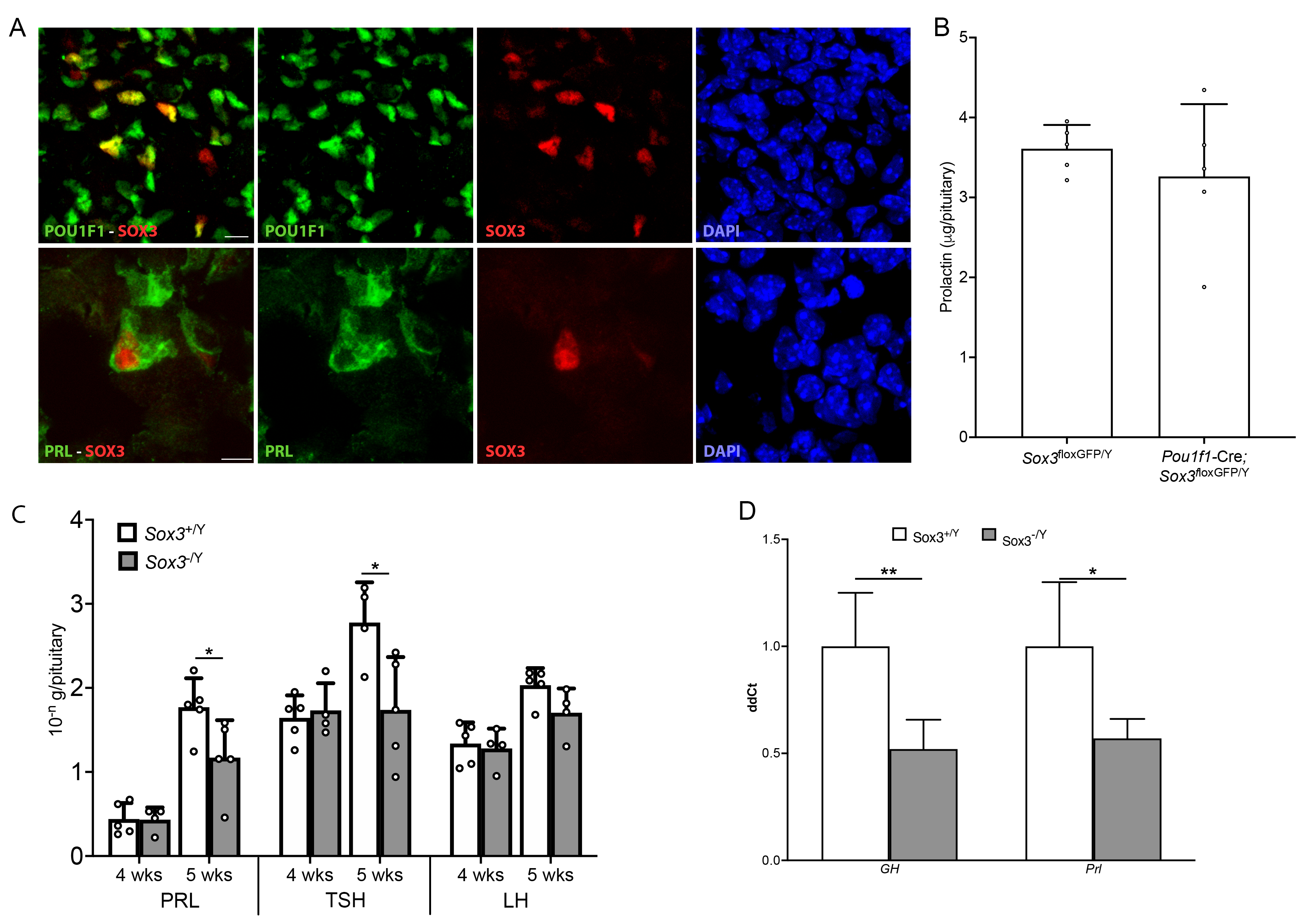

Supplement: S1 Fig — (A) Fluorescent immunolabelling for SOX3, POU1F1 and Prolactin (PRL) on 2-month old Sox3+/Y anterior pituitaries. Scale bars represent 10μm. (B) PRL contents in 2-month Sox3floxGFP/Y and Pou1f1-Cre; Sox3floxGFP/Y. (C) PRL, TSH and LH contents in 4 weeks and 5 weeks Sox3+/Y and Sox3-/Y pituitaries. Concentrations on the Y axis: LH, 10-6g per pituitary; PRL, 10-6g per pituitary; TSH, 10-7g per pituitary. *: p<0.05. (TIF) [file pgen.1011395.s001.tif]

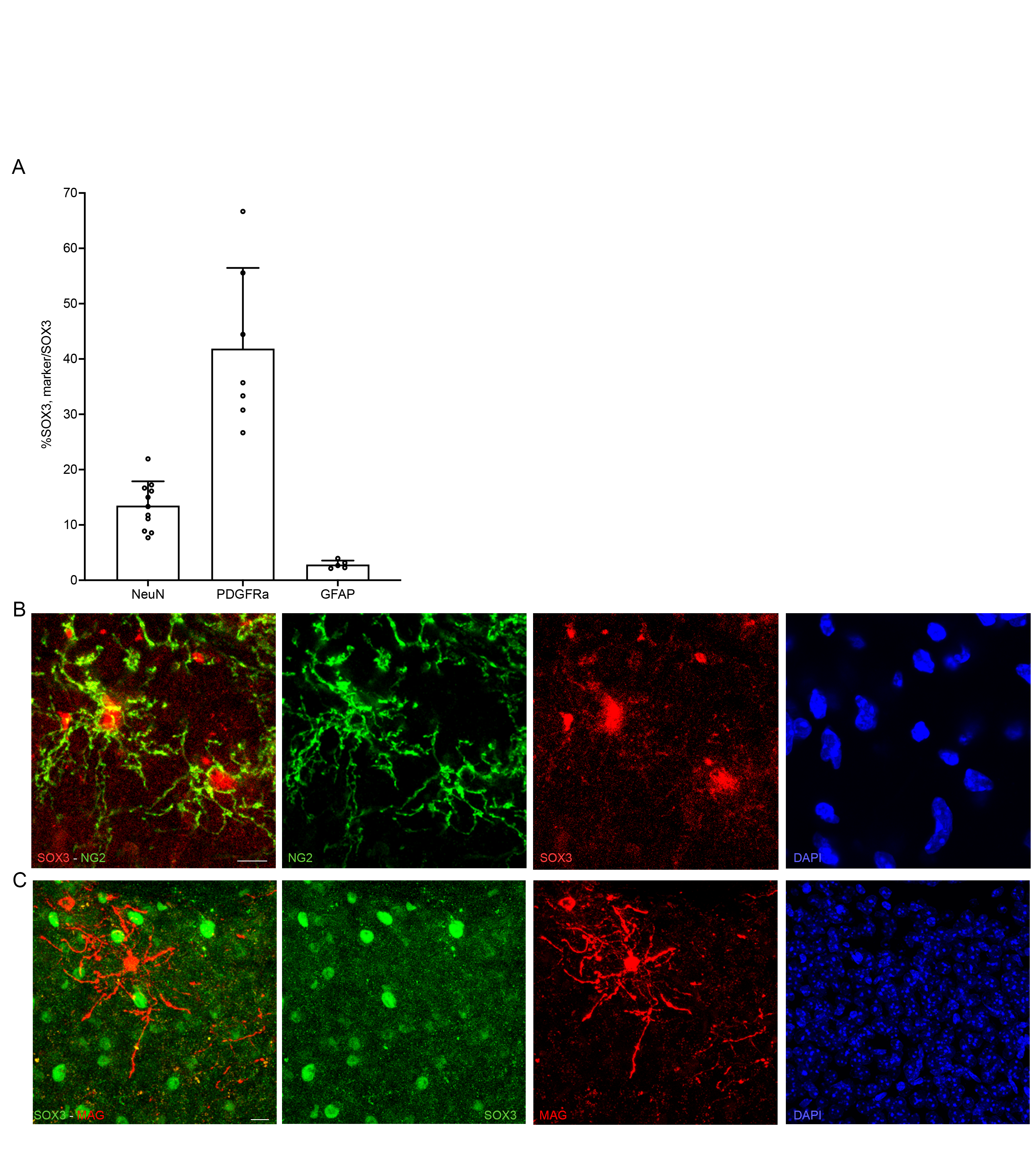

Supplement: S2 Fig — (A) Percentage of SOX3+ve cells in Sox3+/Y hypothalami expressing NeuN, PDGFRa or GFAP. (B-C) Fluorescent immunolabelling for SOX3, NG2 (B) and MAG (C) on 2-month Sox3+/Y MEs. SOX3 is expressed in NG2+ve NG2-glia but not in MAG+ve mature oligodendrocytes. Scale bars represent 10μm. (TIF) [file pgen.1011395.s002.tif]

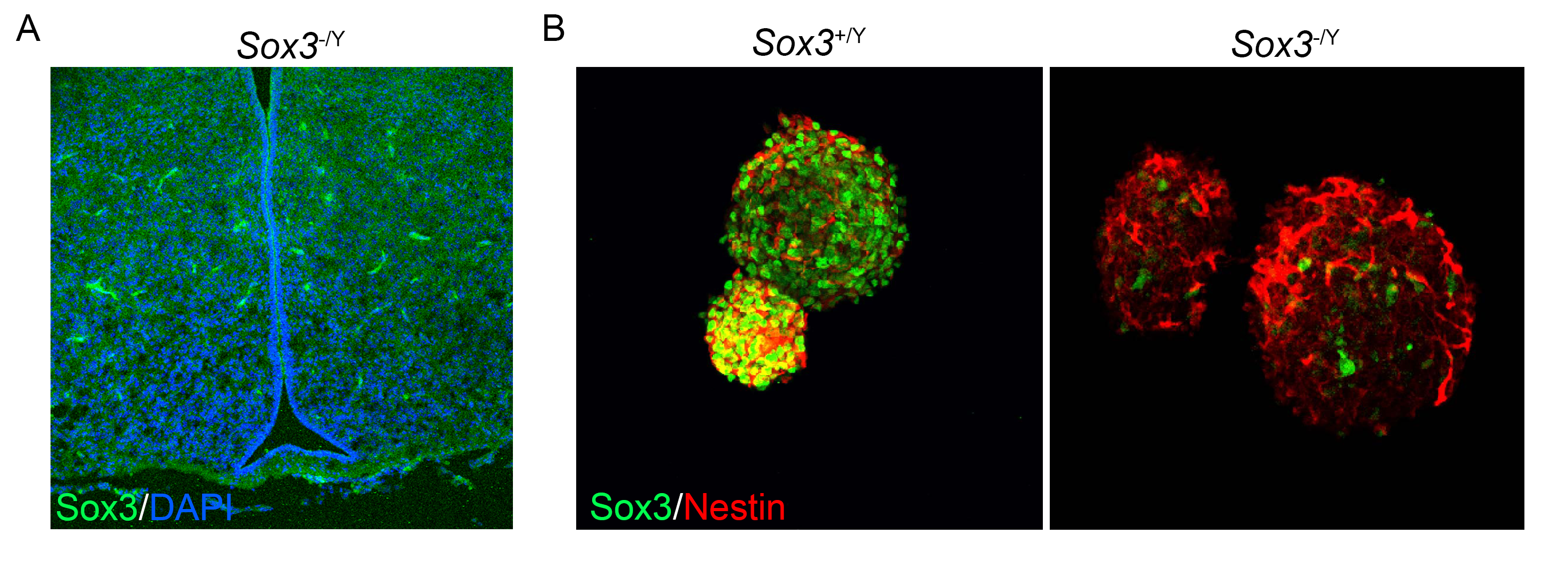

Supplement: S3 Fig — (A) Fluorescent immunolabelling for SOX3 on 2-month old Sox3-/Y hypothalamic. (B) Fluorescent immunolabelling for SOX3 and Nestin on Sox3+/Y and Sox3-/Y neurospheres. Note the absence of SOX3 in Sox3-/Y spheres. (TIF) [file pgen.1011395.s003.tif]

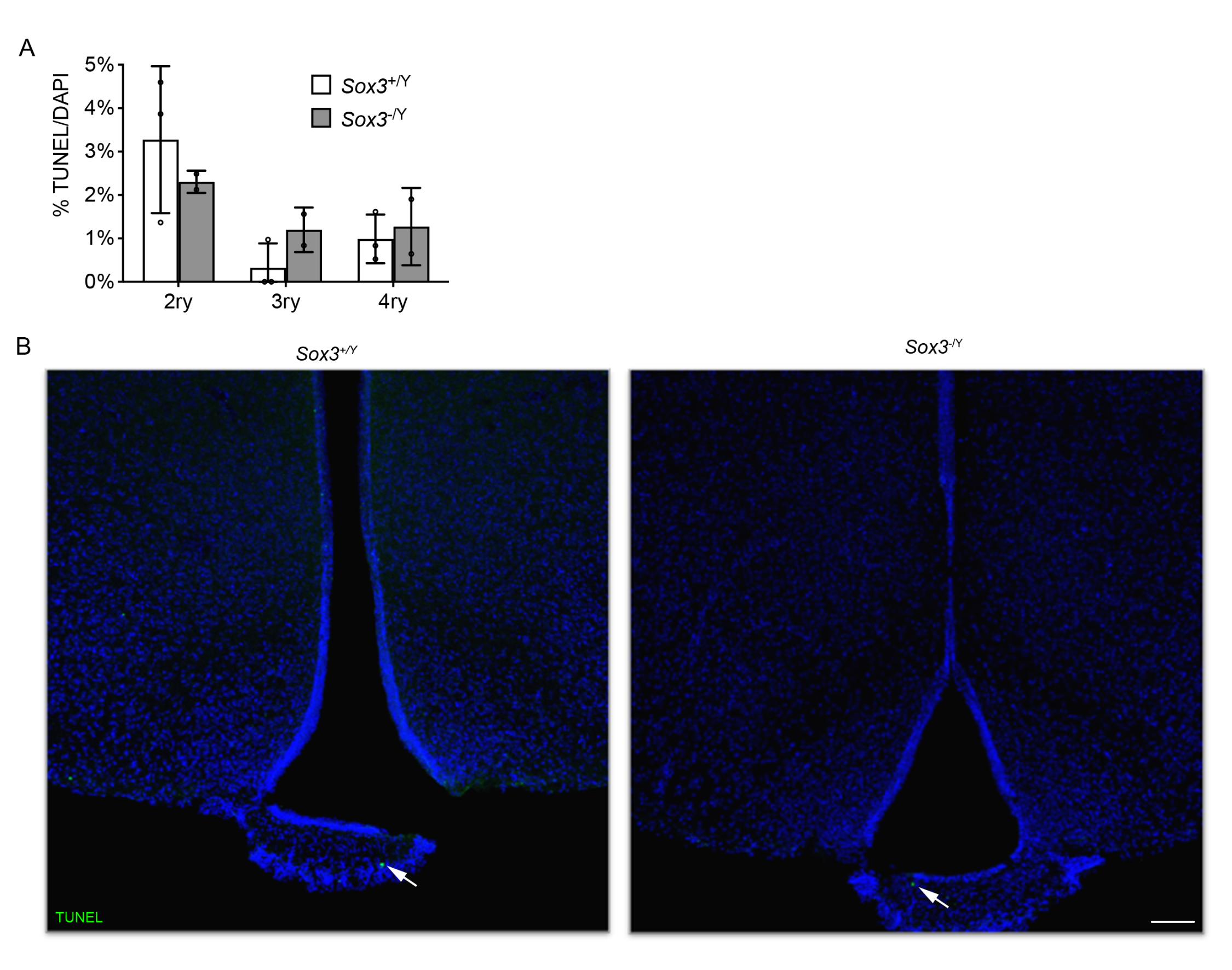

Supplement: S4 Fig — (A) Percentage of TUNEL+ve cells in Sox3+/Y (white columns) and Sox3-/Y (grey columns) of monolayer cultures derived from adult animals at secondary, tertiary, and quaternary passages. No significant difference is observed. (B) TUNEL staining on Sox3+/Yand Sox3-/Y 2-month hypothalami. Arrows point to TUNEL+ve cells. Very few positive cells are present in both samples. Scale bar represents 100μm. (TIF) [file pgen.1011395.s004.tif]

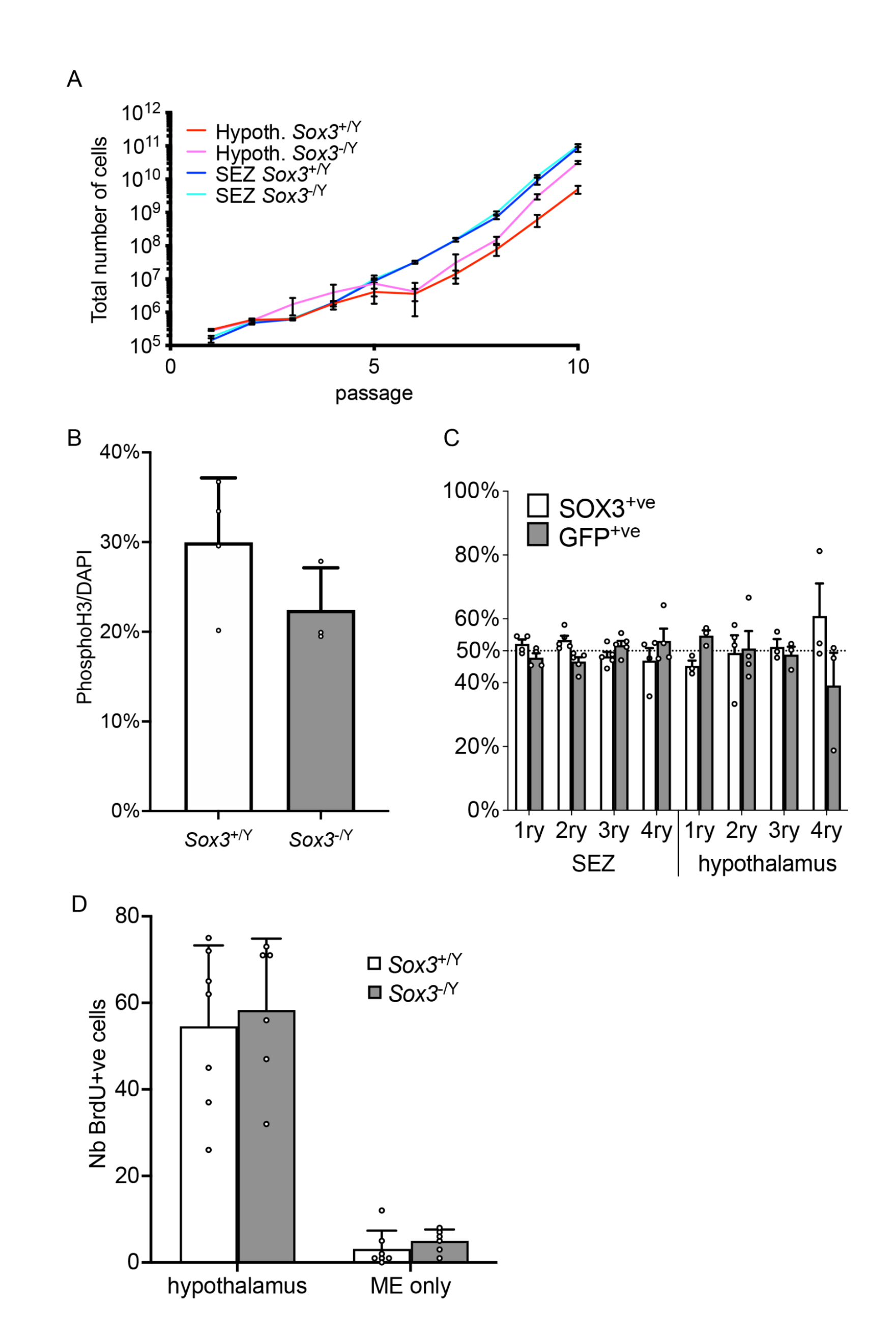

Supplement: S5 Fig — (A) Total number of cells counted after each passage from hypothalamic derived (red/pink) or SEZ derived (dark and light blue) NSCs from ten-day old Sox3+/Y (dark blue/red) and Sox3-/Y mice (light blue/pink). Sox3-/Y NSCs from pups self-renew normally. (B) Percentage of phospho-histone H3+ve cells in secondary progenitor monolayer cultures originated from ten-day old Sox3+/Y and Sox3-/Y hypothalami. Sox3-/Y progenitors proliferate normally. (C) Percentage of neurospheres containing mostly SOX3+ve cells (white columns) or mostly GFP+ve cells (grey columns) derived from ten-day old Sox3+/ΔGFP SEZ or hypothalami. SOX3-ve, GFP+ve progenitors are maintained in these cultures from young females. (D) Number of BrdU+ve cells in one-week Sox3+/Y (white columns) and Sox3-/Y (grey columns) hypothalamic parenchymas and MEs. The number of BrdU+ve cells is similar in Sox3-/Y compared to Sox3+/Y samples. (TIF) [file pgen.1011395.s005.tif]

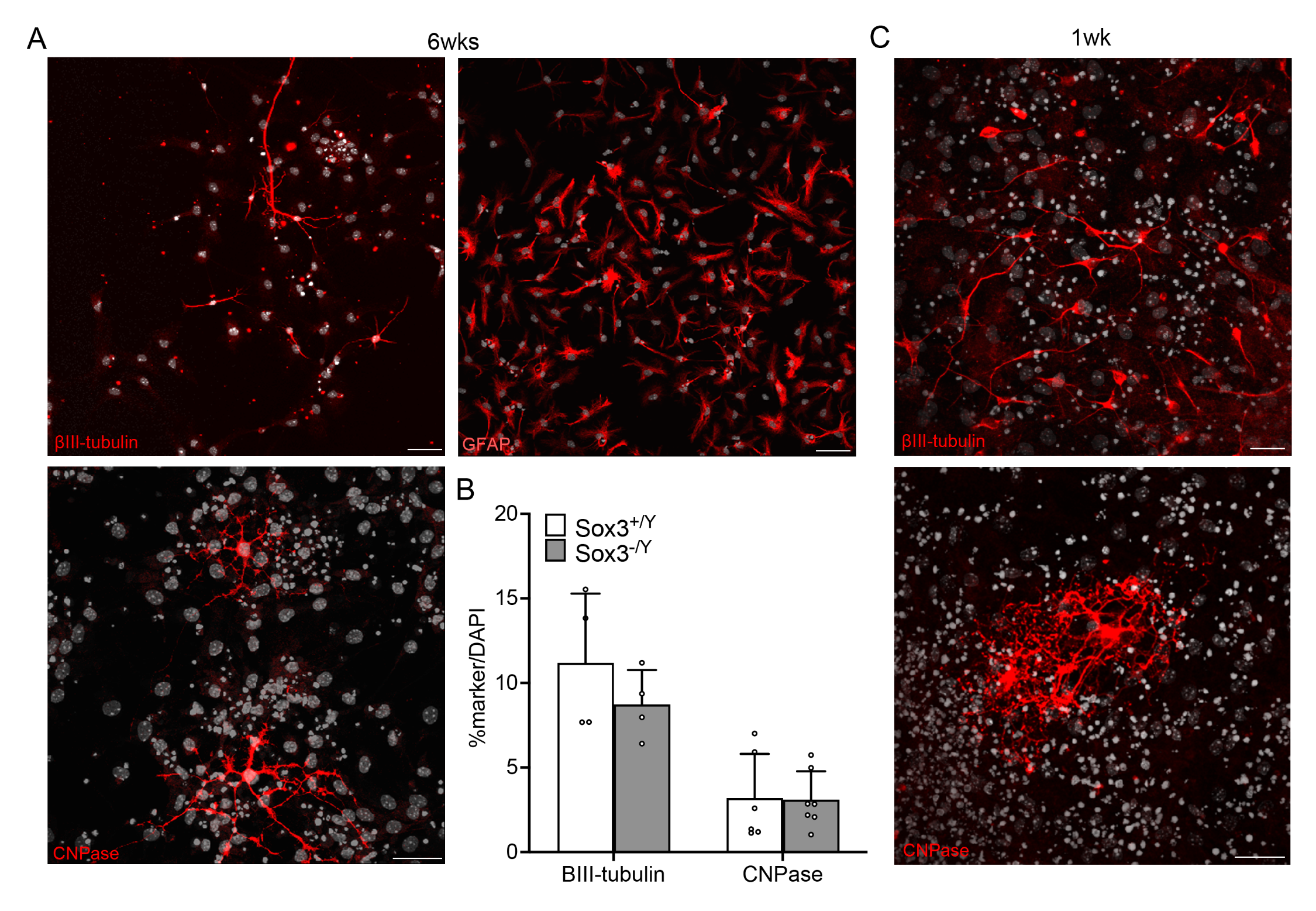

Supplement: S6 Fig — (A-C) Fluorescent immunolabelling for GFAP (astrocytes), βIII-tubulin (neurons), CNPase (oligodendrocytes) on 6-weeks (A) and 1-week (C) Sox3+/Y hypothalamic differentiated progenitor monolayers. (B) Percentage of cells differentiated into βIII-tubulin+ve neurons and CNPase+ve oligodendrocytes derived from 6-weeks Sox3+/Y (white columns) or Sox3-/Y (grey columns) hypothalami. Scale bars represent 30μm. (TIF) [file pgen.1011395.s006.tif]

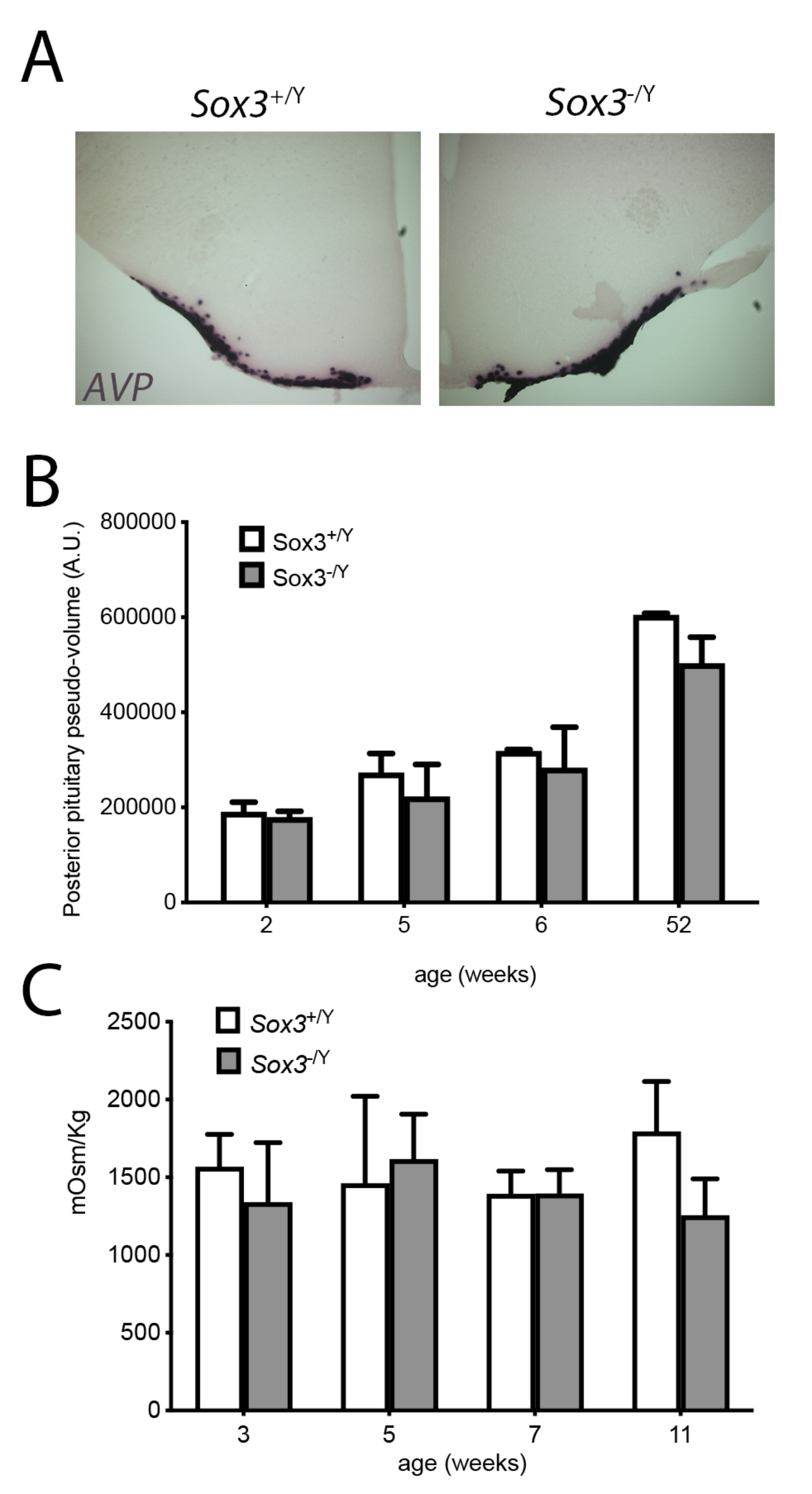

Supplement: S7 Fig — (A) In situ hybridization for AVP on 2-month Sox3+/Y and Sox3-/Y hypothalami. (B) Sox3+/Y and Sox3-/Y posterior pituitary sizes at different post-natal weeks. Sox3-/Y posterior pituitaries are not smaller to that of Sox3+/Y animals. (C) Urine osmolality measurement at different post-natal weeks. (TIF) [file pgen.1011395.s007.tif]

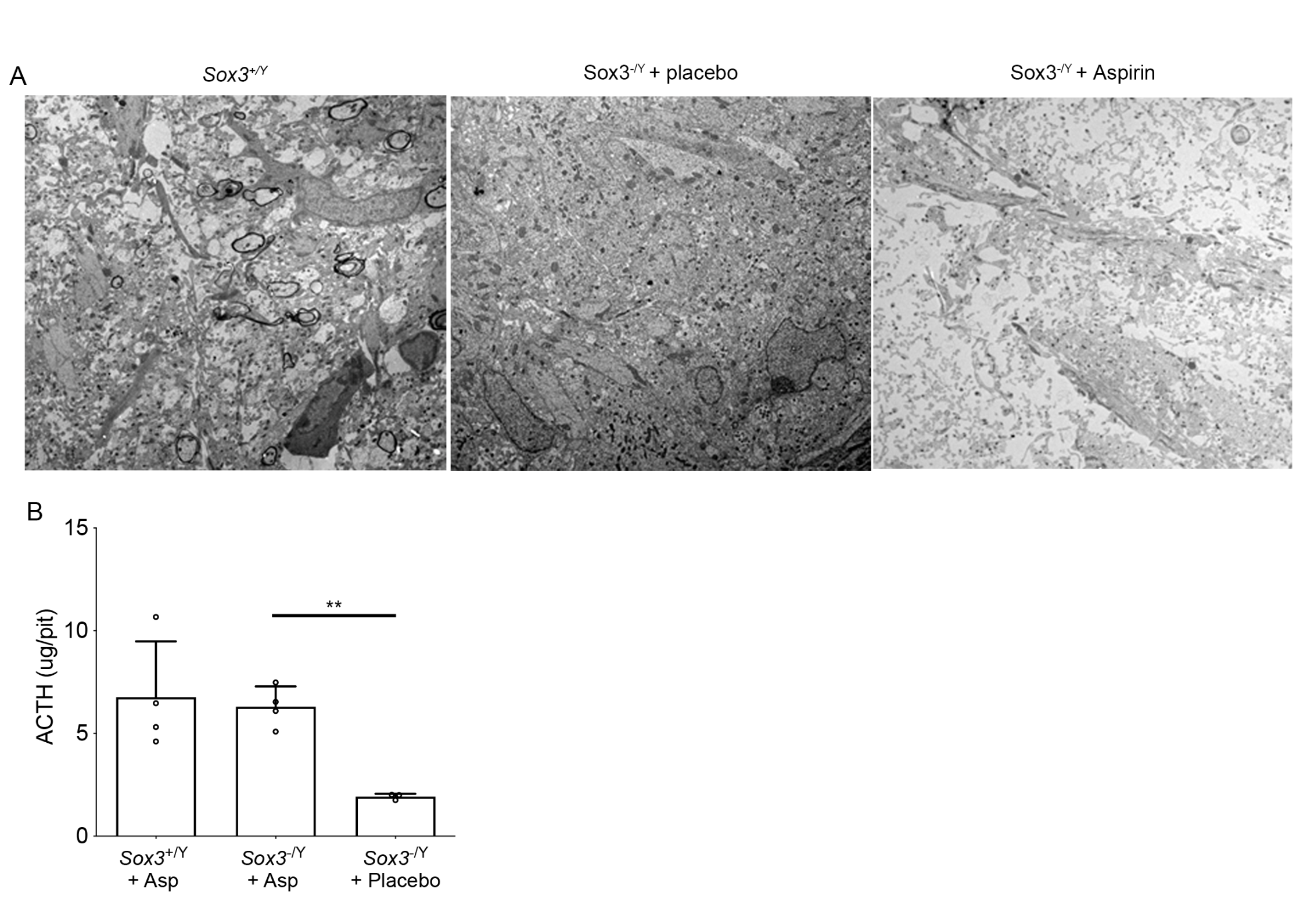

Supplement: S8 Fig — (A) Transmission electron microscopy images of 2-month Sox3+/Y, Sox3-/Y with placebo and aspirin-treated Sox3-/Y MEs. Myelinated axons are missing in aspirin-treated Sox3-/Y MEs. (B) ACTH contents in 2-month Sox3+/Y, Sox3+/Ywith low dose aspirin, Sox3-/Y with low dose aspirin and Sox3-/Y with placebo pituitaries. Note the rescue in ACTH contents in Sox3-/Y mice receiving low dose aspirin. **: p<0.01. (TIF) [file pgen.1011395.s008.tif]

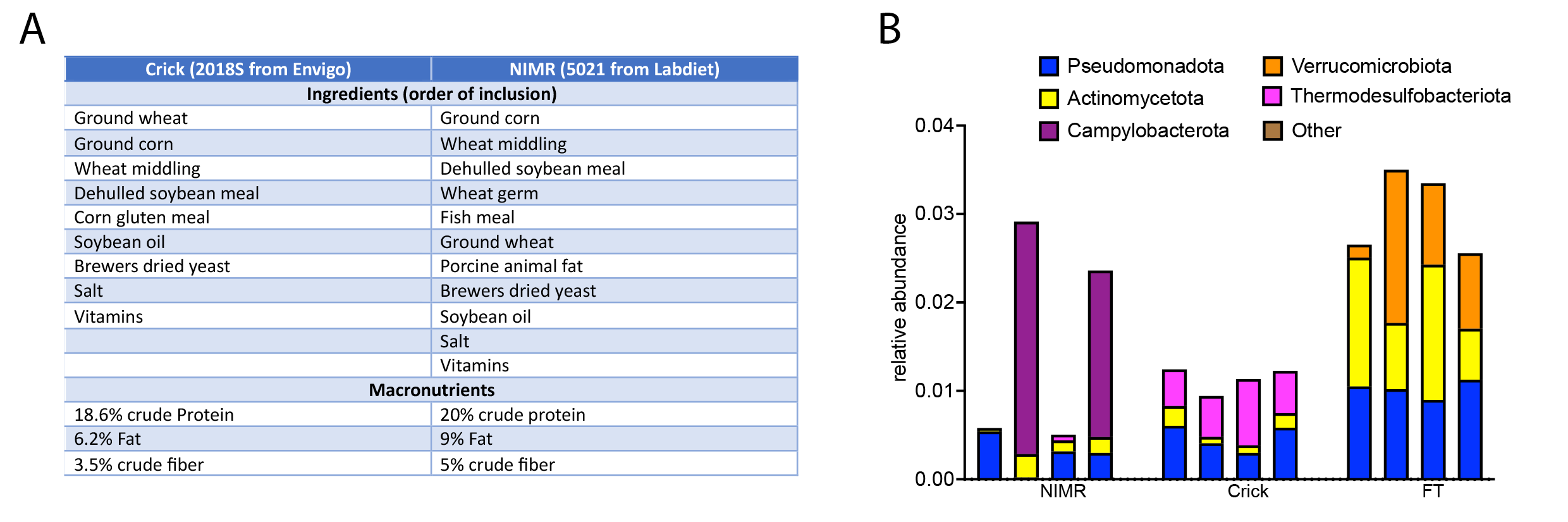

Supplement: S9 Fig — (A) Composition of rodent chow at NIMR and Crick. (B) Abundance of different bacteria phylum in gut microbiota of mice housed at NIMR, Crick or faecal transplanted (FT) focussing only on lowly expression phyla. (TIF) [file pgen.1011395.s009.tif]
