# Supplementary material for: Hypopituitarism in Sox3 null mutants correlates with altered NG2-glia in the median eminence and is influenced by aspirin and gut microbiota
Source: PLoS Genet. 2024 Sep 26;20(9):e1011395. doi: 10.1371/journal.pgen.1011395 (PMC11426531; doi:10.1371/journal.pgen.1011395)
Supplement: Attachment — Submitted filename: Response to reviewers.docx [file pgen.1011395.s010.docx]

Dr. Bruce Hamilton, Academic Editor

Dr Hua Tang, Section Editor

*PLOS Genetics*

Dear Dr Hamilton and Dr Tang,

Re: Resubmission of manuscript reference PGENETICS-D-24-00062

Please find attached a revised version of our manuscript originally entitled “Sox3-null hypopituitarism depends on median eminence NG2-glia and is influenced by aspirin and gut microbiota” to be considered for publication in *PLOS Genetics*.

We have answered all reviewers’ comments, modified our manuscript and added information accordingly, as detailed below. We believe that our manuscript has been much improved by these corrections.

Within the revised manuscript, the revisions are highlighted in yellow. In accordance with the first comment, the title has been revised and is now: “Hypopituitarism in *Sox3* null mutants correlates with altered NG2-glia in the median eminence and is influenced by aspirin and gut microbiota”.

We hope that the revisions of the manuscript and our accompanying responses make our manuscript suitable for publication in *PLOS Genetics*.

Yours sincerely,

Christophe Galichet

On behalf of

Christophe Galichet, Karine Rizzoti and Robin Lovell-Badge

We thank the reviewers for their constructive comments. By taking these into account, we consider that our manuscript has been much improved.

***Reviewer* #*1:***

***Major Comments***

*The authors establish a correlation between hypopituitarism and NG2+ cell alterations in the median eminence. However, a causal relationship is not definitively demonstrated. The interpretation, as well as the title ("depends on"), should be adjusted to reflect this limitation.*

While the weight of evidence makes a strong case for the involvement of NG2-glia in *Sox3* mutant hypopituitarism, we agree that this is not definitively demonstrated. We have therefore modified the title. The new title is:

“Hypopituitarism in *Sox3* null mutants correlates with altered NG2-glia in the median eminence and is influenced by aspirin and gut microbiota.”

*The focus on NG2 glia as the primary mechanism for hypopituitarism may need further justification. Since Sox3 deletion affects the self-renewal of hypothalamic NSCs, particularly tanycytes, another explanation could be an alteration in the tanycyte population's ability to regulate hypothalamic neurohormone release in the ME vasculature.*

We thank the reviewer for this comment. We agree that tanycytes can regulate hypothalamic neuropeptide release at the ME level, although, as far as we are aware, this has only been shown for GnRH and TRH which would not explain the pan-hypopituitarism we see with the *Sox3* mutants. Nevertheless, because we cannot exclude the involvement of tanycytic NSCs in the phenotype, we have modified the discussion accordingly (line 538 - 544) to better reflect this possibility:

“Tanycytes are directly involved in regulating GnRH and TRH release into the blood stream at the ME level (for review: (1-3)), and it is possible that SOX3 plays a role in this, as well as in their self-renewal as we reveal by proliferation and neurosphere assays. However, unless other neurohormones are similarly affected this would not explain the pan-hypopituitarism we see in *Sox3* mutants, moreover, the phenotype and its rescue correlate with alterations in NG2 glia.”

*Consider the role of the Cox-prostaglandin pathway in tanycytic control of neurohormone release, especially given aspirin's effect on NG2-glia. This pathway might contribute to the beneficial effects of aspirin on pituitary function.*

We agree with the reviewer that the Cox-prostaglandin pathway has been described in tanycytes and that the effect of aspirin could be mediated via this pathway. Therefore, to reflect this possibility, we have added the following to the discussion (line 575 -581):

“COX signalling is active in tanycytes. Furthermore, inhibition of the pathway at the ME has been proposed to impair the ovarian cycle through its effect on tanycytes.(4). Therefore, we cannot exclude a tanycytic component of the beneficial effect that low-dose aspirin has on Sox3^-/Y^ hypopituitarism. However, dissecting the relative contribution of tanycytes, NG2-glia and microglia to the phenotype will require new methods that avoid the use of tamoxifen, or of other agents that can themselves affect pituitary function.”

*Provide evidence for the loss of Sox3 expression in Sox3-null mice, both in vivo and in vitro.*

We have generated a supplementary figure (S3 Fig) showing the lack of SOX3 in *Sox3* mutants both *in vivo* and *in vitro*. This is also mentioned in the manuscript (line 228).

“...express GFP, the open reading frame of which replaces that of *Sox3* (Fig 2Hiv) and not SOX3 (S3A-B Fig)”

*Further characterization of pituitary defects could include evaluating pituitary hormone gene expression via in situ hybridization or RT-qPCR.*

We showed that pituitary hormone levels (protein) are reduced in *Sox3* mutants by radio-immunoassay (RIA). We also showed that this can be replicated at the mRNA level using RT-qPCR (Fig 6). However, to make this more explicit for the readers, we also include a sentence (line 155 – 156) and panel in S1 Fig to clarify that reduction of pituitary hormones can be determined by RIA and RT-qPCR.

“... and luteinizing hormone (LH) (S1C Fig; ([26](http://applewebdata://C41BBCF3-3771-4704-8B71-F9C6E75FEEB5#_ENREF_26))) and similar results were obtained when analysing *Gh* and *Prl* gene expression (S1D Fig)”

*Quantify Sox3-expressing cells (line 206-210) to enhance characterization.*

We have now quantified SOX3 expressing cells using neuronal (NeuN), astrocytic (GFAP) and a OPC (PDGFRa) markers. This has been included in supplementary figure 2 and referred to in the body of the manuscript. In agreement with a predominant effect on OPCs in *Sox3* mutants, we find that the majority of SOX3 expressing cells are positive for PDGFRa. Beside these 3 cell types, it is likely that SOX3 is expressed in neuroblasts and during oligodendrocyte differentiation, as shown in the spinal cord (5). (line 215-216)

(A) Percentage of SOX3^+ve^ cells in *Sox3*^+/Y^ hypothalami expressing NeuN, PDGFRa or GFAP.

*Include illustrative microphotographs for Figure 1F and corresponding quantification for Figure 1G.*

We agree that to help the readers, illustrative images and quantification are necessary in Figure 1F and G. These have now been added and the figure legend has been modified accordingly (line 199-206):

(F) *Sox3*^+/Y^ and *Sox3*^-/Y^ TSH^+ve^ cell volumes at 1 week (white columns) and 2 months (grey columns). Insets show 1-week and 9-month *Sox3*^+/Y^ and *Sox3*^-/Y^ pituitaries immunolabelled for TSH. TSH^+ve^ cell volumes increase with age in *Sox3*^+/Y^ but remain constant in *Sox3*^-/Y^ mice. (G) Transmission electron microscopy images of 2-week and 6-month *Sox3*^+/Y^ and *Sox3*^-/Y^ anterior pituitaries. The bar chart shows the number of secretory granules in *Sox3*^+/Y^ and *Sox3*^-/Y^ pituitaries at 2 weeks (white columns) and 6 months (grey columns). Note that fewer secretory granules are present in 6-month *Sox3*^-/Y^ anterior pituitaries. Scale bar: 2µm.

*: p<0.05; **: p<0.01; ***: p<0.001

*In Figure 2G, the statement about Rax expression needs adjustment due to apparent differences in microphotographs. Ensure consistency between images and conclusions.*

We rephrased the sentence in the text (line 221-223) to better reflect the description of the images in Figure 2G. However, we believe that it does not affect our conclusion regarding the emergence of tanycytes.

“*Rax* expression pattern between *Sox3*^-/Y^ and *Sox3*^+/Y^ animals is similar, albeit possibly slightly reduced in *Sox3*^-/Y^ hypothalami, suggesting that deletion of *Sox3* does not affect tanycyte emergence (Fig 2G)”

It is likely that the morphology of the section itself explains the apparent thicker ependymal layer observed in *Sox3*^+/Y^ animals. In agreement with this, the initial number of neurospheres we obtain at early passages is comparable between *Sox3* mutant and wild-type samples suggesting that tanycyte numbers are not affected by the loss of SOX3.

*For Figure 3C, where BrdU incorporation in tanycytes is quantified, clarify the method of tanycyte identification and provide an illustrative microphotograph. Indicate any statistical differences on the bar graph.*

We agree with the reviewer that little indication was given on how the BrdU^+ve^ tanycytes were identified. We have now given the information in the Materials and Methods section of the manuscript (Line 716). We also indicated the non-significance of the difference seen on the graph (Fig 3).

“a and b tanycytes were determined by their relative location within the hypothalamus.”

*In Figure 3F, the upper panel's Sox2 green signal appears non-specific. This should be addressed for clarity.*

We agree with the reviewer that the SOX2 signal in Figure 3F may show some non-specific signal. We have now replaced the image with one that is clearer.

***Minor comments***

*Specify that hypothalamic NSCs mentioned (line 132, 211, 247) correspond to tanycytes for clarity.*

We have now made this clear in lines 134, 219 and 258.

*Clarify if "SEZ" refers to the SEZ of the lateral ventricles.*

This has now been changed (line 237, 251 and 648)

*Early mention of the Sox3 gene location on the X-chromosome (line 239) would clarify the naming convention for transgenic males ("Sox3-/Y").*

We agree with the reviewer and have now included this information in the introduction (line 105).

“In both humans and mice, mutations in *SOX3/Sox3*, which is located on the X chromosome, are associated with hypopituitarism.”

*In Figure 1D, indicate the location of the anterior lobe on the microphotographs for clarity.*

An outline around the anterior pituitary has been drawn. The figure legend has also been modified accordingly (line 193-195):

(D) *Sox3^+/Y^* and *Sox3^-/Y^* anterior pituitary sizes at different post-natal weeks. Insets show 2-month *Sox3^+/Y^* and *Sox3^-/Y^* H&E-stained sections where the anterior pituitary is outlined. *Sox3^-/Y^* anterior pituitaries are smaller post-weaning. Scale bar: 200µm.

***Reviewer #2***

***Major Comments***

*The figures/figure panels do not seem to be presented in order throughout the manuscript which is potentially confusing for readers. We highly recommend presenting the figures/figure panels in order to avoid confusion.*

We believed that we have kept the order throughout the manuscript. For some results we must refer to figures either appearing later or already mentioned. However, we made sure that this is kept to a minimum.

*It may be a good idea to include a figure panel with a schematic of how the FT experiment was performed. It would be helpful to more immediately know if this was a single fecal transplant or multiple doses of the bacteria.*

A schematic representation of how the FT experiment was performed has been added to Figure 6

*It is disappointing that the FT experiment only included the rederivation of the NIMR microbiome in germ-free mice in the Crick facility. Ideally this should also included transplant of Crick microbiome in germ-free mice as a control as well. Importantly, what type of diet was administered to germ-free mice? The Crick diet or the NIMR diet? Far more methodological clarity is necessary here to truly determine whether this is microbiome driven.*

We understand the concern of the reviewer. Following our move to the Crick facility, our main issue has been the recovery of the *Sox3*^-/Y^ panhypopituitarism. We carefully examined all potential differences and decided to try faecal transplants (FT) since the diets between the two facilities were drastically different (Supplementary figure 9 and below). It became clear that performing the transplants ourselves was not efficient, even after testing several antibiotics to deplete the existing microbiome prior to transplantation. The remaining alternative was a germ-free rederivation followed by FT. However, the costs were high (around £10,000 per round) and we thus decided to limit this to the experiment that mattered to pursue our analysis of the phenotype: attempting to recover the panhypopituitarism by restoring the NIMR microbiome.

We also understand and agree with the reviewers that a more detailed description on how the experiment was performed would be beneficial to the readers. We therefore modified the section relating to gut microbiota in Materials and Methods (see below).

*I would highly recommend repeating the experiment with these appropriate controls to delineate the true contribution of microbiome to their phenotype. Otherwise, the authors should mention that this is a severe limitation of their study and how more rigorous testing is required to determine the contribution of the microbiome vs. Diet.*

We understand that the experiments performed are not as extensive as they could have been, and now highlight the experimental limitations in the Discussion (line 610 – 619):

“To confirm the contribution of the microbiome to the Sox3 null phenotype, experiments would have to be performed in more rigorous settings in which mice under germ-free conditions would be faecal transplanted with either NIMR or Crick materials. While we have not formally tested the effect of the different diets on the microbiome, GH-deficient FT mice and their progeny have been kept on the Crick diet suggesting that the phenotypic effect is primarily due to microbiome differences. Furthermore, certain bacteria within the gut microbiota could trigger inflammation throughout the body (for review (6)). Further experiments need to be undertaken to determine the level of inflammation in the different settings and whether the beneficial effect of aspirin is due to its anti-inflammatory action. “

*For Panel A in Fig 6, the smallest sections of the pie charts are difficult to identify. It looks like Firmicutes (green) appear twice. Improving the image quality will enhance the clarity of this panel. Overall, we find pie charts to be a poor method for conveying compositional differences. Alternatively, the authors could use a stacked taxonomic barplot to display these data, which would make it easier to compare across groups. In addition, to convey individual data, we recommend that the stacked bar plots to be for individual mice with the different groups clustered together. Importantly, for a more holistic view of the gut microbiota composition differences, ordination plots like PCA or PCoA should be used to visualize the overall similarity between microbial communities from different groups, with statistical differences conveyed with PERMANOVA. As it stands the description of composition differences, and whether they are truly statistically different is quite incomplete.*

We agree with the reviewer than a more holistic view of gut microbial composition is important, and we thank the reviewer for the comments and recommendations. We have replaced the pie charts by individual stacked bar plots grouped by cohort (Fig 6B and S9B Fig) as well as including a PCA plot (Fig 6C). We believe that this improves the visualisation of the gut microbiota composition across the different animal groups.

*For Panel B in Fig 6, it is now recognized that firmicutes/bacteroidetes ratio is primarily driven by the amount of fat in the mouse diet, whereas earlier microbiome studies seem to imply that there was something more significant about this particular phylum ratio. Though the authors are illustrating compositional differences between their cohorts, it may be a good idea to use log-ratios to convey this information which is a more accurate way of depicting this data and convey a bit more information about compositional differences (see PMID 31222023, which does a better job of explaining the importance of log-ratios).*

We have now modified Fig6B to better reflect the reviewer’s comments. Fig 6D now represents the log-ratio between firmicutes and Bacteroidetes.

*For Panel C in Fig 6, could you please provide additional details on how alpha diversity was measured in your study? Specifically, it would be helpful to know which alpha diversity metric(s) you employed (e.g., Shannon index, Simpson index, Chao1, observed species) and any software or analytical tools used to calculate these metrics.*

We understand that the details provided for the measurement of alpha diversity were limited. As requested, we have now incorporated the additional details in Fig 6E and in the text and in the gut microbiota section of the materials and methods (line 432-433 & 702).

“At the species level and using Shannon index which evaluates both abundance and richness of species, we found that the alpha... “

“Data was analysed with OneCodex online software.”

*The discussion provides a good overview of potential pathways through which the gut microbiota might influence brain function and development. However, further detailing the specific mechanisms, perhaps by elaborating on how these pathways specifically interact with NG2-glia or affect hypopituitarism, would deepen the reader's comprehension of the underlying biology.*

We believe that we have already adequately addressed this point and added further discussion through our responses to the reviewers’ comments. As the reviewer mentioned previously, we have not found the mechanism by which gut microbiota is affecting the phenotype in *Sox3*^-/Y^ animals, but this affect was only discovered by chance (due to the relocation of our institute), and it was never the major aim of the study. We mention in the discussion that further experiments need to be performed to determine the mechanism and how different pathways may interact with NG2-glia.

*The “fecal transplant and gut microbiota sequencing” under the Materials and methods section needs more elaboration: Specify the process used for rederiving Crick mice into germ-free conditions. Include any specific procedures, conditions, or equipment used to ensure the mice were maintained germ-free.
Detail the method used for homogenizing NIMR fecal materials. Mention the volume of PBS used and any specific conditions (e.g., temperature, time) for homogenization.
Clarify whether the oral gavage was performed once or if it was part of a repeated schedule. Also, indicate the volume administered to each mouse.
Clarify length of treatment prior to phenotyping.
Specify the whole-genome sequencing process, including the sequencing platform used, the coverage depth aimed for, and any specific library preparation protocols followed.*

We agree with the reviewer that a more detailed description on how the experiment was performed would be beneficial to the readers. We have therefore re-written the paragraph related to the “fecal transplant and gut microbiota sequencing” under the Materials and Methods section to incorporate the comments and recommendations mentioned by the reviewer (Line 693 – 704).

“*Crick mice were rederived via embryo transfer into germ-free recipient females and maintained in isolators under germ-free conditions at Taconic Biosciences facility (Taconic Biosciences). Frozen NIMR faecal materials were homogenised in 100ul PBS at room temperature for 1 minute. Freshly prepared supernatant (around 75 – 80ul) was then administered by oral gavage once per day for 2 days (TruBIOME, Taconic). Fecal transplanted (FT) mice were sent to the Crick facility for further breeding and maintenance. The FT mice were kept on Crick diet. For sequencing, freshly collected or snap-frozen faecal materials from 4 different animals in each group were incubated in stabilisation buffer and subjected to shotgun whole genome sequencing with read depth of 2 millions paired-end reads (Transnetyx). Data was analysed with OneCodex online software. Gut microbiota datasets are publicly available at the Sequence Read Archive (SRA) of the National Library of Medicine (PRJNA1113545).*”

*Lines 411-413: The authors note the absence of hypopituitarism in Sox3-null mice after relocating to the Crick Institute. However, it is unclear whether the mice were directly transported from the previous facility, or were they part of a new breeding cohort established at Crick? If the former, could the authors specify the timeframe within which these changes manifested? Understanding the temporal dynamics of how dietary and environmental modifications impact gut microbiota composition—and subsequently, hypothalamic functions—is crucial for deepening our insights into the interplay between diet, gut microbiota, and host physiological responses.*

The way the mice were relocated to the Crick (rederivation by embryo transfer) is now mentioned in the text to help the reader understand the change of gut microbiota (line 420):

“Following our relocation and the rederivation by embryo transfer of our animals to the Francis Crick institute (Crick) from the National...”

*There needs to be a statement in the text about how many mice from each group were included in this study. Based on graphs it appears that 4-5 mice were used. If so, this should be made explicit in the methods. Also if any mice were excluded from analysis, this should be noted as well. Additionally in lines 418 - 419, the authors should state the number of mice whose gut microbiota was compared from the previous and current institution.*

This has been made clearer in the text (line 429), in the Materials and Methods (line 700) and in Figure 6 B, C, D and E:

“We analysed 4 animals for each group and found substantial differences in gut microbiota composition (Fig 6A, B and S9B Fig).”

“For sequencing, freshly collected or snap-frozen faecal materials from 4 different animals in each *group...*”

**Minor Comments:**
*There are instances where "SOX3" is used when the discussion seems to be specifically about mice. For example in line 105. In these cases, for strict adherence to naming conventions, it would be clearer to use "Sox3" when referring to findings specifically in mice. This nuanced approach to naming helps clarify which species' gene is being discussed, providing clear differentiation between human and mouse research findings. It's a subtle distinction but important for scientific accuracy and clarity.*

We have now ensured that the correct nomenclature is being used throughout the manuscript. However, in regard to line 114, we are referring to SOX3 protein and this is the correct nomenclature for mouse and human proteins (see Jackson Labs website for nomenclature or https://biosciencewriters.com/Guidelines-for-Formatting-Gene-and-Protein-Names.aspx).

*In line 139, the abbreviation 'GH' is used without prior definition. The full term 'growth hormone (GH),' provided in the following line, should be introduced at its first occurrence in line 139. Similarly on line 152, PRL, TSH and LH are introduced without prior definition. Additionally on line 153, GHRH is introduced for the first time without prior definition.*

We agree with the reviewer and apologise; these were oversights on our part. We have rectified each in line 141 for GH, line 154 for TSH and LH and in line 157 for GHRH.

*The authors use “Sox3+/Y” or “controls” interchangeably, and “Sox3-/Y” or “Sox3-null” interchangeably throughout the manuscript. To improve clarity for readers, I suggest being consistent with one term.*

To help the readers, we have changed *Sox3*-null to *Sox3*^-/Y^ and controls to *Sox3*^+/Y^ where appropriate throughout the manuscript. The changes are highlighted in yellow in the revised manuscript.

***Have all data underlying the figures and results presented in the manuscript been provided?*** *Large-scale datasets should be made available via a public repository as described in the PLOS Genetics* [*data availability policy*](https://eur03.safelinks.protection.outlook.com/?url=http%3A%2F%2Fjournals.plos.org%2Fplosgenetics%2Fs%2Fdata-availability&data=05%7C02%7C%7Ceafa67c2f745429062b808dc64884ce0%7C4eed7807ebad415aa7a99170947f4eae%7C0%7C0%7C638495783784492997%7CUnknown%7CTWFpbGZsb3d8eyJWIjoiMC4wLjAwMDAiLCJQIjoiV2luMzIiLCJBTiI6Ik1haWwiLCJXVCI6Mn0%3D%7C0%7C%7C%7C&sdata=zLCnLoUSyOlooH2IGkrWRGstfD9RT%2BR5UDCKQ1DZm3k%3D&reserved=0)*, and numerical data that underlies graphs or summary statistics should be provided in spreadsheet form as supporting information.*

*Reviewer #1: Yes*

*Reviewer #2:* ***No:*** *microbiome data*

The latter was an oversight and the gut microbiota datasets have now been deposited into Sequence Read Archive (SRA) of the National Library of Medicine (PRJNA1113545).

REFERENCE

1.	Prevot V, Dehouck B, Sharif A, Ciofi P, Giacobini P, Clasadonte J. The Versatile Tanycyte: A Hypothalamic Integrator of Reproduction and Energy Metabolism. Endocr Rev. 2018;39(3):333-68.

2.	Parkash J, Messina A, Langlet F, Cimino I, Loyens A, Mazur D, et al. Semaphorin7A regulates neuroglial plasticity in the adult hypothalamic median eminence. Nat Commun. 2015;6:6385.

3.	Muller-Fielitz H, Stahr M, Bernau M, Richter M, Abele S, Krajka V, et al. Tanycytes control the hormonal output of the hypothalamic-pituitary-thyroid axis. Nat Commun. 2017;8(1):484.

4.	de Seranno S, d'Anglemont de Tassigny X, Estrella C, Loyens A, Kasparov S, Leroy D, et al. Role of estradiol in the dynamic control of tanycyte plasticity mediated by vascular endothelial cells in the median eminence. Endocrinology. 2010;151(4):1760-72.

5.	Hoffmann SA, Hos D, Kuspert M, Lang RA, Lovell-Badge R, Wegner M, et al. Stem cell factor Sox2 and its close relative Sox3 have differentiation functions in oligodendrocytes. Development. 2014;141(1):39-50.

6.	Al Bander Z, Nitert MD, Mousa A, Naderpoor N. The Gut Microbiota and Inflammation: An Overview. Int J Environ Res Public Health. 2020;17(20).
