# Supplementary material for: Hypopituitarism in Sox3 null mutants correlates with altered NG2-glia in the median eminence and is influenced by aspirin and gut microbiota
Source: PLoS Genet. 2024 Sep 26;20(9):e1011395. doi: 10.1371/journal.pgen.1011395 (PMC11426531; doi:10.1371/journal.pgen.1011395)
Supplement: Attachment — Submitted filename: response_to_reviewers.docx [file pgen.1011395.s011.docx]

Dr. Bruce Hamilton, Academic Editor

Dr Hua Tang, Section Editor

PLOS Genetics

Dear Dr Hamilton and Dr Tang,

Re: Resubmission of manuscript reference PGENETICS-D-24-00062R1

Please find attached a revised version of our manuscript entitled “Hypopituitarism in *Sox3* null mutants correlates with altered NG2-glia in the median eminence and is influenced by aspirin and gut microbiota” to be considered for publication in PLoS Genetics.

We thank the reviewers for reviewing the manuscript again. We have made minor changes to the manuscript in response to Reviewer 2 as detailed below.

Within the revised manuscript, the revisions are highlighted in yellow.

We hope that the revisions of the manuscript and our accompanying responses make our manuscript suitable for publication in *PLOS Genetics*.

Yours sincerely,

Christophe Galichet

On behalf of

Christophe Galichet, Karine Rizzoti and Robin Lovell-Badge

We thank the reviewers for their constructive comments. By taking these into account, we consider that our manuscript has been improved.

***Reviewer #1:***

***The authors satisfactorily answered Reviewer's comments***

We thank the reviewer for taking the time to review the manuscript again.

***Reviewer #2:***

***Minor Comments***

*I would encourage the authors to mention the diet in their description of the experiment in the results. That is, in lines 434 and onwards: “To test whether changes in gut microbiota underlie rescue of hormonal deficiencies, we performed faecal transplant (FT) from NIMR animals into Crick animals ***, while maintaining the mice on the Crick diet.***” This will help readers understand which variables/aspects of the experiment are being manipulated.*

We thank the reviewer for the comment and have added the following in line 425, in line 436 and in line 640:

“…included the adoption of a free-from animal protein and fish meal diet (Labdiet 5021 and Envigo 2018S at NIMR and Crick respectively - S9A Fig).”

“...animals into Crick animals, while maintaining the mice on Crick diet (Envigo 2018S).”

“Mice had access to food and water ad libitum with diet 5021 (Labdiet) at NIMR and diet 2018S (Envigo) at the Crick.”

*Given a recent paper that states that timing of sample collection could affect microbiome results (PMID 38951660), I would like the authors to confirm that the time of sample collection at NIMR and Crick facility was the same/similar and include additional information about stool collection time to confirm that this is not an artifact of changes in stool collection.*

We thank the reviewer and understand the new paper highlights the timing of sample collection as a parameter for variability. We collected our samples between 10am and 12-noon (3 to 5h in the light cycle). We have added the following into the materials and methods.

“For sequencing, freshly collected or snap-frozen faecal materials collected between 10am and 12-noon (3 to 5h in the light cycle) from 4 different animals in each group were incubated in stabilisation buffer…”
